# Supplementary material for: RSBagging: An ensemble classifier detecting the after-effects of ischemic stroke through EEG connectivity and microstates
Source: PLoS One. 2024 Oct 22;19(10):e0311558. doi: 10.1371/journal.pone.0311558 (PMC11495553; doi:10.1371/journal.pone.0311558)
Supplement: Attachment — Submitted filename: response_letter_plosone.pdf [file pone.0311558.s001.pdf]

Response to the Review

Manuscript Ref No.: PONE-D-24-22084

"RSBagging: An Ensemble Classifier Detecting the after-effects of Ischemic Stroke through EEG Connectivity and Microstates"

Fang Wang, Xueying Zhang, Peng Zhang, Fengyun Hu

First of all, we would like to acknowledge the editor, associate editor and anonymous reviewers for their comments and suggestions. These comments and suggestions are invaluable for us to improve the quality and readability of our manuscript. We have considered these comments and suggestions carefully, and then have revised the paper accordingly. Following the Editor's suggestions, we now resubmit our revised manuscript for your further consideration for publication.

The following are our detailed responses to the editor and reviewers on how we have addressed all their comments and suggestions and what changes we have made accordingly to the original version of our manuscript. All changes are marked in red in our revised manuscript.

---

1 Response to Editor-in-Chief

a) The entire editorial decision letter:

Dear Dr. Wang,

Thank you for submitting your manuscript to PLOS ONE. After careful consideration, we feel that it has merit but does not fully meet PLOS ONE's publication criteria as it currently stands. Therefore, we invite you to submit a revised version of the manuscript that addresses the points raised during the review process.

Please submit your revised manuscript by Oct 24 2024 11:59PM. If you will need more time than this to complete your revisions, please reply to this message or contact the journal office at plosone@plos.org. When you're ready to submit your revision, log on to https://www.editorialmanager.com and select the 'Submissions Needing Revision' folder to locate your manuscript file.

Please include the following items when submitting your revised manuscript: A rebuttal letter that responds to each point raised by the academic editor and reviewer(s). You should upload this letter as a separate file labeled 'Response to Reviewers'. A marked-up copy of your manuscript that highlights changes made to the original version. You should upload this

as a separate file labeled 'Revised Manuscript with Track Changes'. An unmarked version of your revised paper without tracked changes. You should upload this as a separate file labeled 'Manuscript'. If you would like to make changes to your financial disclosure, please include your updated statement in your cover letter. Guidelines for resubmitting your figure files are available below the reviewer comments at the end of this letter.

If applicable, we recommend that you deposit your laboratory protocols in protocols.io to enhance the reproducibility of your results. Protocols.io assigns your protocol its own identifier (DOI) so that it can be cited independently in the future. For instructions see: https://journals.plos.org/plo guidelinesloc-laboratory-protocols. Additionally, PLOS ONE offers an option for publishing peer-reviewed Lab Protocol articles, which describe protocols hosted on protocols.io. Read more information on sharing protocols at https://plos.org/protocols?utm$_{m}edium = editorial - emailutm_{s}ource = authorlettersutm_{c}ampaign = protocols$.

We look forward to receiving your revised manuscript.

Kind regards,

Ramu Anandakrishnan, Ph.D. Academic Editor PLOS ONE

Journal requirements:

When submitting your revision, we need you to address these additional requirements.

1. Please ensure that your manuscript meets PLOS ONE's style requirements, including those for file naming. The PLOS ONE style templates can be found at

https://journals.plos.org/plosone/s/file?id=wjVg/PLOSOne$_{f}ormatting_{s}ample_{m}ain_{b}ody.pdfand$

https://journals.plos.org/plosone/s/file?id=ba62/PLOSOne$_{f}ormatting_{s}ample_{t}itle_{a}uthors_{a}ffiliations.pd$

2. We note that you have indicated that there are restrictions to data sharing for this study. For studies involving human research participant data or other sensitive data, we encourage authors to share de-identified or anonymized data. However, when data cannot be publicly shared for ethical reasons, we allow authors to make their data sets available upon request. For information on unacceptable data access restrictions, please see http://journals.plos.org/plosone/s/data-availabilityloc-unacceptable-data-access-restrictions.

Before we proceed with your manuscript, please address the following prompts:

a) If there are ethical or legal restrictions on sharing a de-identified data set, please explain them in detail (e.g., data contain potentially identifying or sensitive patient information, data are owned by a third-party organization, etc.) and who has imposed them (e.g., a Research Ethics Committee or Institutional Review Board, etc.). Please also provide contact information for a data access committee, ethics committee, or other institutional body to which data requests may be sent.

b) If there are no restrictions, please upload the minimal anonymized data set necessary to replicate your study findings to a stable, public repository and provide us with the relevant URLs, DOIs, or accession numbers. Please see http://www.bmj.com/content/340/bmj.c181.long for guidelines on how to de-identify and prepare clinical data for publication. For a list of recommended repositories, please see https://journals.plos.org/plosone/s/recommended-repositories. You also have the option of uploading the data as Supporting Information files, but we would recommend depositing data directly to a data repository if possible.

Please update your Data Availability statement in the submission form accordingly.

b) *Our Response:*

Thank you for your encouraging comments and suggestions. In our revised manuscript, we have addressed all comments from you and the two reviewers #1, #2. Our point-by-point responses to the comments of reviewers are respectively enclosed in Sections 2 and Sections 3 of this document. We hope that the revisions adequately address the reviewers' concerns and improve the quality of our manuscript.

Regarding the data, we have uploaded the processed data to the link provided in our manuscript. Both the code and data are available for review.

c) Additional Editor Comments:

Dear Dr. Wang,

This study presents an interesting approach for developing a classifier for predicting the after-effect of stroke. However, the reviewers have several questions, comments and concerns that need to be addressed. In addition, to these comments, please address the following: 1. Add a section describing the limitations of this study. 2. Compare the results for the various models using the training dataset versus the test dataset, discuss any differences and the implication for how the models might perform on an independent test set.

Thank you.

d) Our Response:

We have carefully considered your comments and concerns and have made the following revisions:

Limitations of the Study: We have added a new section to the manuscript that outlines the limitations of our study. This section discusses potential sources of bias, the constraints of our dataset, and the generalizability of our findings.

Comparison of Model Performance: To compare the performance of different models, we have added two additional rows in Table 2 to provide a clearer comparison. The revised manuscript now includes a detailed description of this table in the text.

In addition, all results presented in the manuscript are derived from the test dataset. The comparison of all models was conducted using this test set. At the outset of the experiment, 20% of the data was randomly allocated as an independent test dataset to ensure an unbiased evaluation of model performance.

Encl: *Responses to Reviewers #1, #2.*

. . . to be continued on the next page for Response to Reviewer #1

2 Response to Review #1

The paper is well-written and the ensemble method combining SVM classifiers trained on balanced training subsets through undersampling produced impressive classification performance. Some comments to improve readability of the paper:

Our Response: *We sincerely appreciate the time and effort you have dedicated to reviewing our manuscript. Your insightful comments have been invaluable in enhancing the quality and readability of the paper. We have carefully considered all of your feedback and implemented substantial revisions accordingly. Below, we provide detailed responses to each comment.*

Comment 2.1: Please state the shape of the data (how many total features per input, since there are pairwise connectivity metrics for each pair, is it quadratic on the electrode locations?)

Our Response: *Thank you for your comment. For each connectivity feature, the input shape is 190, representing the pairwise connections from 20 electrodes. We have updated the manuscript to include the input shape, as detailed below:*

> *"These matrices are computed across frequencies from 1 Hz to 45 Hz and then averaged before being input into the classifiers. Each connectivity matrix has a shape of 190."*

Comment 2.2: It appears, building individual classifiers on balanced subsets through undersampling is the main reason behind the classifier performance. Please provide a discussion on what would happen if a similar undersampling method is used to modify methods like Random Forest or XGBoost.

Our Response: *Thank you for your suggestion. XGBoost uses a stacking architecture, where each layer adjusts for the errors made in the previous predictions. Due to this structure, undersampling cannot be directly incorporated into the XGBoost framework. Similarly, Random Forest also faces challenges in integrating undersampling, as it relies on bootstrapping techniques for creating multiple decision trees, making the direct application of undersampling difficult.*

Comment 2.3: Please make Figure 2 more comprehensible. For example, in step 3, a pair of signals was introduced, but in step 4, these terms were not used.

Our Response: *Thank you for your valuable feedback. We have revised Figure 2, particularly step 3, to improve clarity. The description in step 3 has been updated to "Time series from each electrode," and the reference to the "pair of signals" has been removed accordingly to ensure consistency and comprehensibility in step 4.*

Comment 2.4: Please elaborate on the classification scheme and provide examples of topographic similarity for microstate clustering.

Our Response: *Global Explained Variance (GEV) is used as a measure of how similar each EEG sample is to the microstate prototype. We have extracted GEV as a microstate parameter to serve as input to the classifier. The relevant data and code, including GEV, have been provided in the data and code link included in the manuscript.*

. . . to be continued on the next page for Response to Reviewer #2

3 Response to Review #2

Comment 3.1: There should be some discussion/analysis on time from stoke to EEG as a confounding variable, as well as if there were multiple strokes separated by time as there might have been some degree of recovery after the initial stroke that could influence the results.

Our Response: *Thank you for your insightful comment. Unfortunately, detailed information regarding multiple stroke occurrences and the time from stroke to EEG was not recorded in our dataset. As a result, we have not conducted a longitudinal analysis in this study. We recognize this as a limitation of our research and will address it in the "Limitations" section of the revised manuscript. In future work, we plan to incorporate time and recovery-related factors to further refine the analysis. The limitations in the manuscript is as below:*

> *"Despite the promising results of our EEG-based stroke classification, several limitations should be acknowledged. First, the dataset lacks detailed information on patients with multiple strokes, and the absence of longitudinal data prevents us from accounting for the effects of stroke recovery on brain activity, which may introduce confounding variables. Additionally, the lack of information on neurological recovery between strokes limits our ability to analyze how recovery trajectories affect EEG signals. Future studies should incorporate longitudinal data and more comprehensive medical histories, including stroke timing and frequency, to address these gaps. Finally, while our findings are insightful, the generalizability of the results would benefit from a larger, more diverse dataset. Future research should aim to validate these findings across different stroke subtypes and recovery stages with a broader population."*

Comment 3.2: When comparing the different models, it would be helpful to have a graph or table directly comparing the results of existing models vs the new RSBagging model. The current presentation obfuscates a direct comparison and makes it difficult.

Our Response: *Thank you for your suggestion. To make the comparison between models clearer, we have added the results of the RSBagging model to Table* **??** *for direct comparison. Additionally, we have included the following description in the manuscript:*

> *"The last two rows of Table* **??** *present the best performance of our RSBagging model, using microstate features (with a cluster number of 16) and the PPC connectivity measure, respectively."*

Comment 3.3: There are some small errors, such as SGD not being defined, and other acronyms like COH, PLV are defined multiple times.

Our Response: *Thank you for pointing out these issues. We have addressed them as follows: 1. We have replaced 'SGD' with 'Stochastic Gradient Descent' since it appears only once in the manuscript. 2. We have removed the redundant definitions of COH (Coherence) and PLV (Phase Locking Value) from the "Nonparametric Methods" subsection, ensuring that these terms are defined only once in the introduction section of the manuscript. We appreciate your attention to these details and have made the necessary corrections.*

Comment 3.4: The line "The RSBagging classifier addresses the classification of imbalanced datasets in two mainly steps:..." should say main steps.

Our Response: *Thank you for your feedback. We have revised the sentence to "main steps" as suggested.*

Comment 3.5: Table 1 has inconsistent space formatting for the numbers in the rows, particularly with "middle coma" and "deep coma" having a space after the plus and minus, and a lack of space after a slash.

Our Response: *Thank you for pointing out the inconsistency in the space formatting in Table 1. We have corrected the formatting to ensure consistent spacing for all numbers, particularly addressing the spaces after the plus/minus signs and slashes.*

Comment 3.6: In the results and discussion section, this part does not match Table 1, which lists 23 subjects with bilateral motor disturbance: "Therefore, the stroke subject number in this section is 105 stroke patients subjects including 22 subjects with bilateral motor disturbance, 37 subjects with left motor disturbance, and 46 subjects with right motor disturbance."

Our Response: *Thank you for your careful review. We acknowledge the mistake and have made the necessary corrections. The sentence now reads:*

> *"Therefore, the stroke subject number in this section is 106 stroke patients, including 23 subjects with bilateral motor disturbance, 37 subjects with left motor disturbance, and 46 subjects with right motor disturbance."*

Comment 3.7: This is also incorrect, and should say 47 subjects had a stroke in the right brain: "The dataset in this section contains 135 stroke subjects including 36 subjects with bilateral side stroke in the brain, 52 subjects with stroke in their left brain and 47 subjects with stroke in the left brain."

Our Response: *Thank you for pointing out this error. We have corrected the sentence as follows:*

> *"The dataset in this section contains 135 stroke subjects, including 36 subjects with bilateral stroke, 52 subjects with stroke in the left brain, and 47 subjects with stroke in the right brain."*

Comment 3.8: This is also incorrect, and should say 36 subjects with bilateral brain side: "We complete two classification tasks: subjects with left brain (52) versus subjects with right brain (47), subjects with single brain side (99) versus subjects with bilateral brain side (52)."

Our Response: *Thank you for your feedback. We have corrected the sentence as follows:*

> *"We completed two classification tasks: subjects with left brain stroke (52) versus subjects with right brain stroke (47), and subjects with single brain side stroke (83) versus subjects with bilateral brain side stroke (36)."*

We appreciate your help in identifying this mistake.

Comment 3.9: This should have a number for consistency: "...stroke with left brain (52) vs stroke with the right brain."

Our Response: *We agree with your suggestion and have added the missing number for consistency in the revised manuscript.*

Comment 3.10: There should be a space here "...higher than98

Our Response: *Thanks for your reminder. We have corrected this in the manuscript to ensure proper spacing.*

Comment 3.11: This link to the data is incorrect: https://github.com/linda-edward/RSBagging/data.

Our Response: *We apologize for the incorrect link. We have revised the description of the data link in the manuscript as below:*

> *"The code as well as the data, including the extracted features and labels used in this study, are available at https://github.com/linda-edward/RSBagging."*

========END OF DOCUMENT========
